# The Soluble Fms-like Tyrosine Kinase-1 Contributes to Structural and Functional Changes in Endothelial Cells in Chronic Kidney Disease

**DOI:** 10.3390/ijms232416059

**Published:** 2022-12-16

**Authors:** Annika Schulz, Carolin Christina Drost, Bettina Hesse, Katrin Beul, Marcus Brand, Giovana Seno Di Marco

**Affiliations:** Department of Internal Medicine D, University Hospital Münster, 48149 Münster, Germany

**Keywords:** Fms-like tyrosine kinase-1 (sFlt-1), chronic kidney disease (CKD), endothelial dysfunction, endothelial stiffness

## Abstract

Endothelial cells are a critical target of the soluble Fms-like tyrosine kinase-1 (sFlt-1), a soluble factor increased in different diseases with varying degrees of renal impairment and endothelial dysfunction, including chronic kidney disease (CKD). Although the mechanisms underlying endothelial dysfunction are multifactorial and complex, herein, we investigated the damaging effects of sFlt-1 on structural and functional changes in endothelial cells. Our results evidenced that sera from patients with CKD stiffen the endothelial cell cortex in vitro, an effect correlated with sFlt-1 levels and prevented by sFlt-1 neutralization. Besides, we could show that recombinant sFlt-1 leads to endothelial stiffening in vitro and in vivo. This was accompanied by cytoskeleton reorganization and changes in the endothelial barrier function, as observed by increased actin polymerization and endothelial cell permeability, respectively. These results depended on the activation of the p38 MAPK and were blocked by the specific inhibitor SB203580. However, sFlt-1 only minimally affected the expression of stiffness-sensitive genes. These findings bring new insight into the mechanism of action of sFlt-1 and its biological effects that cannot be exclusively ascribed to the regulation of angiogenesis.

## 1. Introduction

Soluble Fms-like tyrosine kinase 1 (sFlt-1) is a naturally occurring vascular endothelial growth factor (VEGF) antagonist with recognized antiangiogenic properties [1]. The clinical significance of elevated circulating sFlt-1 was first described for preeclampsia, the primary renal complication of pregnancy [2,3]. Yet circulating sFlt-1 levels are increased in different diseases with varying degrees of renal impairment, including chronic kidney disease (CKD) [4,5,6,7,8], given that these levels are mainly negatively correlated with the estimated glomerular filtration rate (eGFR), a measure of renal function [4,5,6,7,8,9].

Serum from renal patients displayed a robust antiangiogenic activity in the chorioallantoic membrane assay, which was prevented by removing sFlt-1 from the samples or supplementation with excess VEGF [4,10]. In addition, circulating sFlt-1 levels correlate with the presence of biomarkers of endothelial dysfunction in these patients [4,11,12]. Notably, such biomarkers are generally associated with a dysfunctional endothelium, suggesting that endothelial structural changes may coexist with impaired endothelial function [13,14].

Endothelial cells are undoubtedly a critical target of sFlt-1 [4,15]. Dysregulation of endothelial cells, including changes in the actin cytoskeleton, reduced proliferation, migratory capacities, and impairment of the barrier function, contributes to the pathophysiology and progression of many disease states and plays a crucial role in interorgan crosstalk [14,16]. In the present paper, we explained that excess sFlt-1 stiffens the endothelial cell cortex, reflecting a series of molecular changes structurally and functionally affecting endothelial cells [17,18,19]. These findings bring new insight into the mechanism of action of sFlt-1 and its biological effects that cannot be exclusively ascribed to the regulation of angiogenesis in CKD and beyond.

## 2. Results

### 2.1. Sera from Patients with CKD and Excess sFlt-1 Stiffen Endothelial Cell Cortex

**Figure 1:**
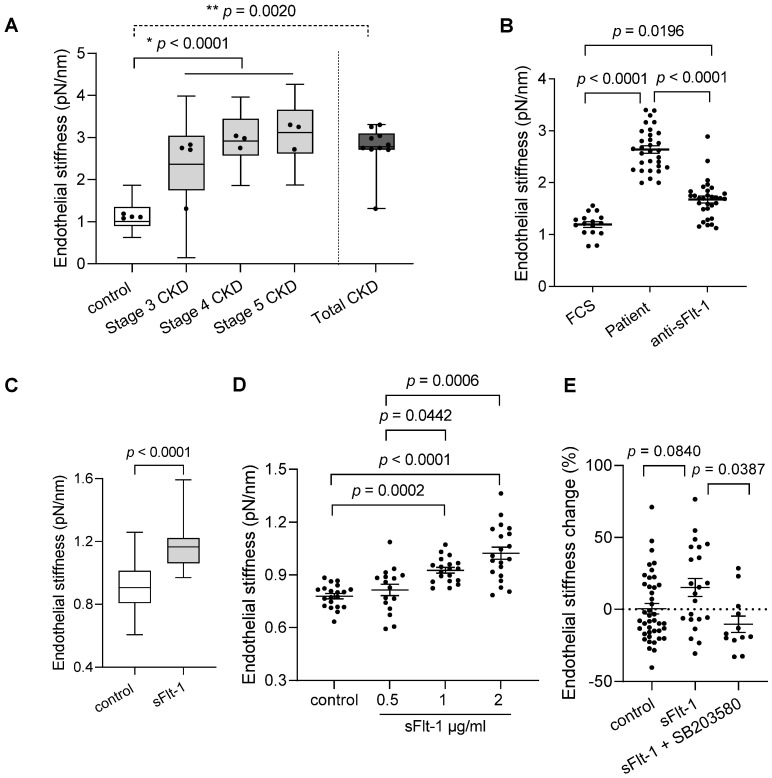
Chronic kidney disease patient sera and excess sFlt-1 stiffen endothelial cell cortex. (**A**) Endothelial stiffness of EA.hy926 cells was measured by atomic force microscopy (AFM) after 24 h of incubation with controls’ and patients’ sera. The values obtained (20–50 values/patient) were grouped according to the stage of CKD (stages three–five), plotted as box and whiskers, and were used for comparison among all three stages and controls (* *p* < 0.0001). Mean values obtained from each patient (dots) were used for comparison between Total (all patients) and controls (** *p* = 0.0020). (**B**) The graphic represents the effect of sFlt-1 neutralization by incubation with a specific antibody followed by immunoprecipitation in protecting cells against patient serum-induced stiffness. Values represent 15–30 measurements/treatments based on two independent experiments. Fetal calf serum (FCS) was used as a reference value (control). (**C**) Aortae from mice exposed to continuous in vivo administration of recombinant sFlt-1 (300 ng/h; N = 3) or control protein (IgG-Fc, 300 ng/h; N = 3) for three days were isolated and analyzed ex vivo by AFM. All values obtained (20–29 values/mouse) were plotted as box and whiskers and used for comparison between groups. Mean values obtained from individual mice are represented as dots. (**D**) Dose-response curve of human recombinant sFlt-1 (0.5–2 µg/mL) incubated with EA.hy926 cells for 24 h. Control cells were treated with control protein (2 µg/mL). (**E**) Endothelial stiffness of primary HUVECs was measured by AFM 24 h after incubation with sFlt-1 or control protein (2 µg/mL). Coincubation with the SB203580 (10 µM), a specific p38 MAPK inhibitor, protected cells from stiffening upon sFlt-1 treatment. Results are expressed as mean ± SEM.

The baseline characteristics of the study participants are given in Table 1. A small cohort of ten patients was divided into three stages of CKD (stages three–five), given that sFlt-1 levels increased with advancing stages. Four healthy subjects were used as controls. Atomic force microscopy of EA.hy926 cells incubated with sera from patients with CKD for 24 h showed increased stiffness of the endothelial cell cortex in an eGFR- and sFlt-1-dependent way, as shown in Figure 1A and Table 1, respectively. The removal of sFlt-1 from serum samples by immunoprecipitation using a specific antibody protected against endothelial stiffening (Figure 1B), thereby demonstrating the implication of sFlt-1.

To further explore the effects of sFlt-1 on the stiffness of the endothelial cell cortex, i.e., endothelial stiffness, we used recombinant sFlt-1 both in vivo and in vitro, thus avoiding confounders present in the human sera as exemplified in Table 1 (endothelial dysfunction markers/risk factors). First, ex vivo analysis in isolated aortae of mice exposed to elevated circulating sFlt-1 levels (300 ng/h) showed increased endothelial stiffness in comparison to the aortae of animals receiving control protein (IgG-Fc, 300 ng/h) (Figure 1C). This finding evidences that excess sFlt-1 may alter the mechanical properties of endothelial cells in vivo.

Second, in vitro analysis of EA.hy926 and primary human umbilical vein endothelial cells (HUVECs) treated with sFlt-1 (0.5–2 µg/mL) revealed increased endothelial stiffness in comparison to cells treated with control protein (recombinant IgG-Fc) in a dose-dependent way (Figure 1D). Therefore, the highest dose (2 µg/mL) was used for further experiments using primary HUVECs (Figure 1E). Interestingly, pretreatment with SB203580 (10 µM), a specific p38 mitogen-activated protein kinase (MAPK) inhibitor, abolished the sFlt-1 effects on the endothelial stiffness (Figure 1E), suggesting that activation of p38 MAPK is necessary for endothelial stiffening upon sFlt-1 treatment.

### 2.2. sFlt-1 Treatment Activates the p38 MAPK Stress Response Pathway

**Figure 2:**
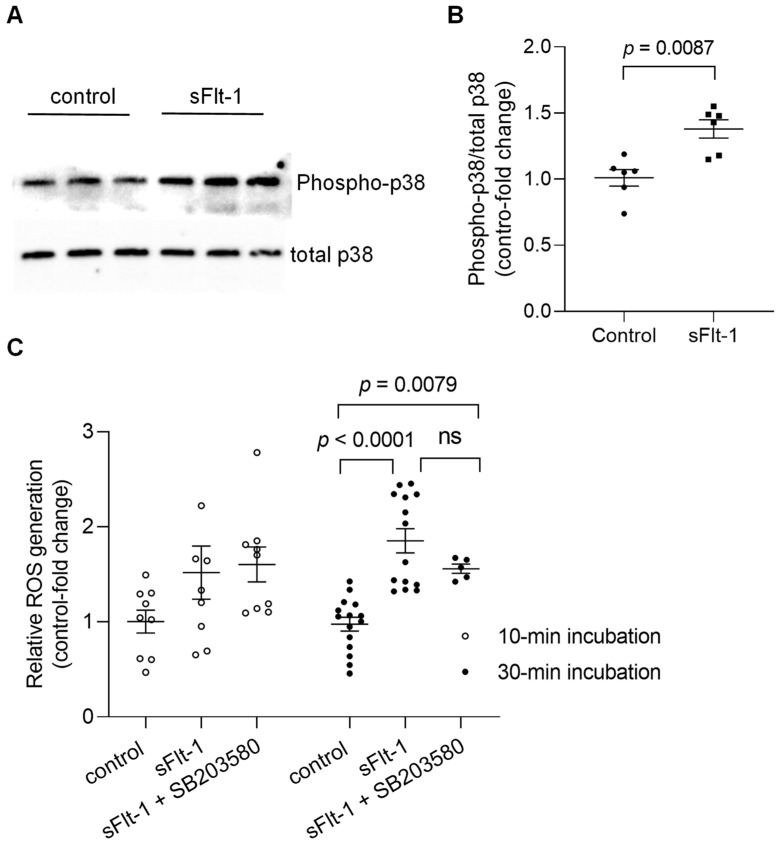
Activation of p38 MAPK and ROS generation upon sFlt-1 treatment. (**A**) Representative Western blotting image of phosphorylated (phospho) and total p38 MAPK 10 min after sFlt-1 treatment. (**B**) Relative densitometric analysis based on two independent experiments. (**C**) ROS was detected by measuring the fluorescence of CellROX Deep Red, a cell-permeant ROS sensor, in cells treated with sFlt-1 for 10 and ~30 min in the presence or not of SB203580 (10 µM). Data are given as fold-change relative to control and expressed as mean ± SEM. MAPK, mitogen-activated protein kinase; ns, not significant; ROS, reactive oxygen species.

Immunoblotting of primary HUVECs confirmed the activation by phosphorylation of p38 MAPK 10 min after incubation with recombinant sFlt-1 (2 µg/mL) (Figure 2A,B). In addition, treatment of endothelial cells with sFlt-1 for 30–45 min resulted in an increased production of reactive oxygen species (ROS) as detected by an increase in CellROX Deep Red fluorescence, a cell-permeant ROS sensor (Figure 2C). Statistically significant alterations in ROS production could not be detected upon shorter incubation time (~10 min). Furthermore, pretreatment with SB203580 (10 µM) had no significant effect on sFlt-1-induced oxidant production at any time point tested.

### 2.3. sFlt-1 Induces Changes in the F-Actin Distribution and Disturbs the Barrier Function of Endothelial Cells

**Figure 3:**
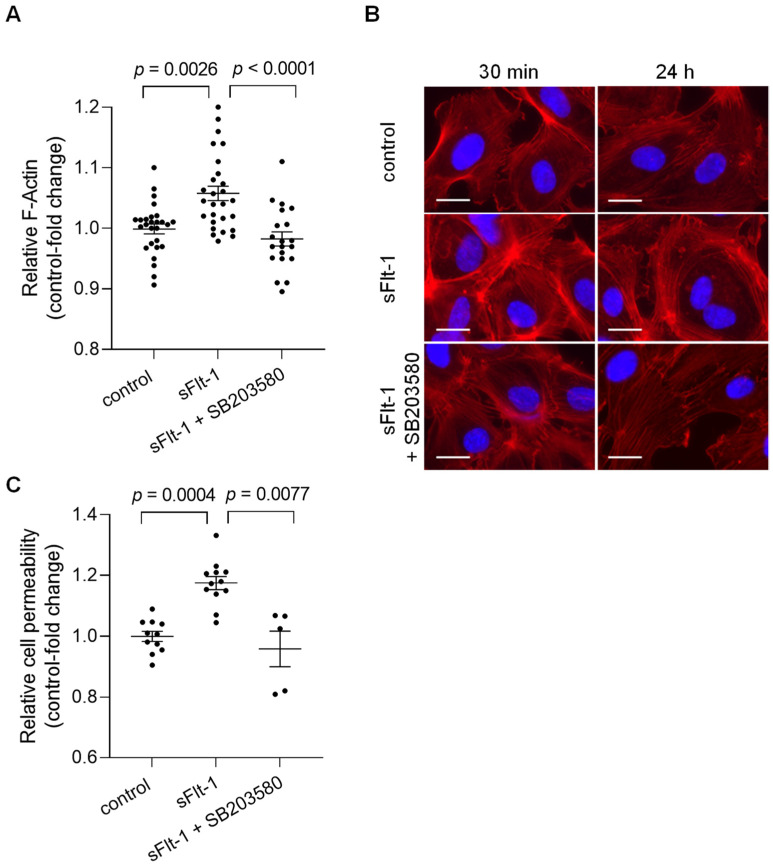
Excess sFlt-1 affects the actin cytoskeleton and disturbs the endothelial barrier function. Primary HUVECs were treated with sFlt-1 or control protein (2 µg/mL) for 24 h in the presence or not of SB203580 (10 µM). (**A**) F-actin was quantitatively assessed by measuring phalloidin fluorescence intensity using a microplate reader. (**B**) Changes in F-actin distribution in cells treated with sFlt-1 for 30 min and 24 h were assessed by immunofluorescence microscopy. Scare bar = 20 µm (**C**) Endothelial cell permeability, as a measure of barrier function, was determined by the passage of streptavidin-HRP through the endothelial monolayer seeded on transwell units (0.4 μm pore). Data are given as fold-change relative to control and expressed as mean ± SEM. HRP, horseradish peroxidase; SB203580, specific p38 MAPK inhibitor.

Alterations in the mechanical properties of endothelial cells, including stiffness of the endothelial cortex, are generally associated with cytoskeletal rearrangements [20]. To determine potential changes in overall cell morphology, actin polymerization was evaluated using phalloidin staining, which detected filamentous actin (F-actin). Quantitative measurements of the fluorescence intensity revealed an increase in total polymerized F-actin in HUVECs treated for 24 h with sFlt-1 (2 µg/mL) compared to cells treated with control protein (2 µg/mL) (Figure 3A). Changes in F-actin distribution could also be observed under sFlt-1 treatment as early as 30 min of incubation, as shown by immunofluorescence images in Figure 3B. Preincubation with SB203580 (10 µM) for 30 min minimized these effects (Figure 3A,B).

Such structural changes can directly affect endothelial cell physiological processes, causing, for example, barrier dysfunction [20]. HUVECs cultivated in transwell inserts were treated with sFlt-1 or control protein (2 µg/mL) for 24 h, and permeability was determined by measuring the passage of streptavidin-horseradish peroxidase across the endothelial monolayer. Excess sFlt-1 significantly increased relative endothelial cell permeability, while p38 MAPK inhibition protected against this effect (Figure 3C).

### 2.4. sFlt-1 Only Slightly Affects Mechanosensitive Genes in HUVECs

**Figure 4:**
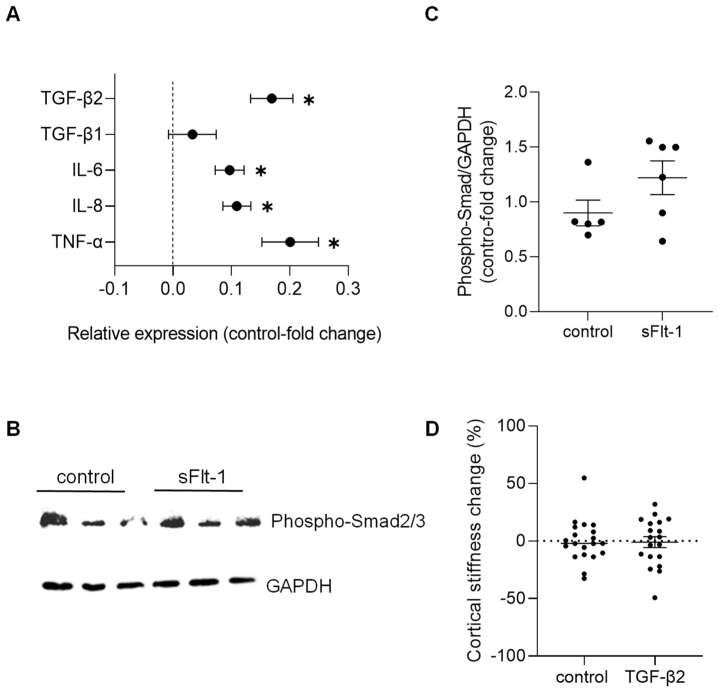
Gene expression and factors associated with excess sFlt-1. Primary HUVECs were treated with sFlt-1 or control protein (2 µg/mL) for 24 h. (**A**) Gene expression analysis was measured by real-time PCR. Results are log-transformed. (**B**) Representative Western blotting image of phosphorylated Smad2/3, the major effector of the TGF-β pathway. GAPDH was used as a loading control. (**C**) Relative densitometric analysis based on two independent experiments. (**D**) Lack of effects of TGFβ2 on endothelial stiffness. Primary HUVECs were treated with recombinant TGFβ2 (10 ng/mL) or vehicle for 24 h, and stiffness was measured by atomic force microscopy. Data are given as fold-change relative to control and are expressed as mean ± SEM. * *p* < 0.05. IL-6, interleukin-6; IL-8, interleukin-8; TGFβ, transforming growth factor-β, TNF-α, tumor necrosis factor-α.

Concerning changes in gene expression profile, we detected only a minimal effect on the expression of stiffness-sensitive genes upon sFlt-1 treatment compared to the treatment with control protein. Real-time PCR results are given in Figure 4A. These results revealed an increase in transforming growth factor-β2 (TGFβ2)—but not TGFβ1—and pro-inflammatory genes (e.g., interleukins 6 and 8, and tumor necrosis factor-α). Even though we observed phosphorylation of Smad2/3—the major effector of the TGF-β pathway—after long-term (24 h) incubation with sFlt-1 (Figure 4B,C), recombinant TGFβ2 (10 ng/mL) did not stiffen the endothelial cell cortex compared to vehicle (Figure 4D).

## 3. Discussion

Circulating sFlt-1 levels are increased in different diseases with varying degrees of renal impairment, including CKD. These levels are most positively associated with proteinuria and inversely correlated with eGFR [4,5,6,7,8,9]. By antagonizing VEGF actions, sFlt-1 directly affects endothelial cell homeostasis [1]. Endothelial dysfunction is common in renal patients and related to adverse clinical outcomes and high mortality [4,21]. In the present study, we investigated the effects of excess sFlt-1 on endothelial stiffness, a structural and functional marker of endothelial cell dysfunction.

We found that sera from patients with CKD stiffen the endothelial cell cortex in vitro. This effect was prevented by sFlt-1 immunoprecipitation, strongly suggesting the participation of sFlt-1 in this effect. However, several soluble factors in the patient’s serum might have directly or indirectly contributed to the changes observed in endothelial stiffness. Therefore, to better understand the role of sFlt-1, we decided to use the human recombinant protein for further experiments. Our main findings showed that excess sFlt-1 leads to endothelial stiffening in vitro and in vivo. This was accompanied by increased F-actin polymerization and reorganization and changes in the endothelial barrier function, as observed by increased endothelial cell permeability. These results depended on the activation of the p38 MAPK and were blocked by the specific inhibitor SB203580. However, sFlt-1 only minimally affects the expression of stiffness-sensitive genes.

Endothelial stiffness reflects the structural and functional properties of the endothelium, including cytoskeleton rearrangement, reduced nitric oxide bioavailability, and enhanced endothelial turnover [17,18,22]. The structure and mechanical properties of the cortical cytoskeleton and the actin mesh beneath the plasma membrane are mainly affected by the actin polymerization state, as actin polymerization stiffens the cortical actin web [18,23]. Endothelial cells take advantage of these mechanical properties to ensure the integrity of the cell monolayer in a quiescent state and adequately respond to physiological and pathophysiological challenges [19,20]. Interestingly, endothelial nitric oxide (NO) release is directly associated with the degree of endothelial stiffness. A soft cortex favors the release of NO, while a stiff one reduces this release and favors vasoconstriction [22]. From a pathophysiological view, a sustained stiffening of the endothelial cortex may contribute to developing endothelial dysfunction and vascular diseases.

Furthermore, many extracellular stimuli are converted into specific cellular responses by activating MAPK signaling pathways [24]. The p38 MAPK is essential in cell stress responses and cytoskeleton reorganization [24,25,26]. Interestingly, p38 MAPK mediates the endothelial cell stiffening in response to intercellular adhesion molecule-1 cross-linking or neutrophil adhesion, as shown by Wang and Doerschuk [25]. Herein, we have demonstrated that p38 MAPK inhibition prevented the F-actin polymerization and the endothelial stiffening under our conditions, suggesting that this pathway may be involved in modulating the actin cytoskeleton rearrangements upon sFlt-1 treatment. To note, changes in F-actin distribution can be observed as early as 30 min of incubation, indicating a rapid effect of sFlt-1 and minimizing the participation of other factors (e.g., gene/protein expression).

Besides, sFlt-1 also significantly affected the endothelial barrier function because high sFlt-1 increased endothelial cell permeability. Even though our experimental design did not precisely delineate the nature of permeability changes (paracellular versus transcellular pathways), increased polymerization and contraction of cortical actin have proven consequences in controlling endothelial permeability [20,27]. Cell–cell junctions might not have withstood the increased mechanical forces generated by endothelial stiffening, favoring intercellular gaps formation [28]. Besides, we do not exclude the involvement of other components of the endothelial monolayer, e.g., endothelial glycocalyx, in regulating endothelial permeability under sFlt-1 treatment [27,29,30]. Further studies will be needed to address this issue. Of note, sFlt-1 did not increase permeability to a comparable extent as excess VEGF usually does, i.e., after about a twofold increase when using a similar assay [31,32,33]. VEGF is a potent vascular permeability factor, and sFlt-1 has been described as a protective agent against its effects [31,34,35,36]. However, in a scenario of increased circulating sFlt-1 and normal-to-decreased VEGF levels—as described in renal pathologies, such as CKD—sFlt-1, could be a relevant contributor to barrier dysfunction [9,37] at least as an important indicator of loss of endothelial integrity. In agreement with the involvement of p38 MAPK on actin rearrangement and, consequently, on barrier dysfunction under sFlt-1 treatment, SB203580 prevented the increase in endothelial permeability.

However, it is still unclear how sFlt-1 activates the p38 MAPK. One hypothesis is that sFlt-1 contributes to an endothelial oxidative stress state, with increased ROS (as shown in Figure 2C) and decreased anti-oxidant capacity due to the impairment of endothelial NO synthase (eNOS) phosphorylation as already described by our group and others [38,39]. Besides, our group has also shown decreased NO generation in an aortic endothelial cell line treated with sFlt-1 [4]. Corroborating our previous findings, several publications directly or indirectly proved the inverse correlation between sFlt-1 and NO formation in animal models and patients and showed the causal role of sFlt-1 in oxidative stress in vitro and in vivo [15,40,41,42]. Among the different mechanisms involved, Sánchez-Aranguren et al. [40] have described early metabolic perturbations and alterations in mitochondrial bioenergetics in endothelial cells—but not in trophoblasts—under sFlt-1 treatment. Unfortunately, only a trend toward an increased ROS production could be detected upon shorter incubation time (~10 min) with sFlt-1 in our study, probably due to the high variability of the values and technical limitations of the assay employed (recommended incubation time > 30 min). However, as the pretreatment with SB203580 did not interfere with sFlt-1 effects at any time point, we can speculate that p38 activation may occur downstream of ROS formation.

Unexpectedly, sFlt-1 did not cause major changes in the expression of stiffness-sensitive genes that appeared to be upregulated, at least in the context of subendothelial stiffness [43]. We observed a slight but significant increase in TGFβ2 expression, which, in combination with the phosphorylation of Smad2/3 proteins, suggested the presence of an activated TGFβ2 pathway under excess sFlt-1 conditions. TGFβ2 modulates the extracellular matrix metabolism, and increased subendothelial matrix stiffness seems to control its activity in HUVECs [43]. Yet, TGFβ2 did not interfere with the mechanical properties of HUVECs under our conditions, excluding the involvement of TGFβ2 in sFlt-1-mediated endothelial stiffening. The upregulation of pro-inflammatory cytokines might reflect the increased ROS/oxidative stress burden in sFlt-1 treated cells. Moreover, especially regarding TNF-α, it could add to changes in endothelial cell morphological and biomechanical properties and the development and evolution of endothelial cell dysfunction [44,45,46,47,48].

Herein, sFlt-1 tested in healthy endothelial cells and mice showed direct, although sometimes weak, effects. Interestingly, it is already described that the impact of increased sFlt-1 is exacerbated if there is a pre-existing endothelial dysfunction (e.g., absence of eNOS). Moreover, excess sFlt-1 renders the endothelium more sensitive to other factors [38,49,50,51]. These findings suggest that sFlt-1 as an additional factor can affect the endothelial nanomechanics under CKD conditions, but not the only one.

## 4. Materials and Methods

### 4.1. Endothelial Cell Culture and Treatment Protocol

Primary human umbilical vein endothelial cells (HUVEC; PromoCell, Heidelberg, Germany) were cultured as recommended by the manufacturer using Endothelial Cell Growth Medium containing fetal calf serum (FCS), endothelial cell growth supplement, epidermal growth factor, basic fibroblast growth factor, heparin, and hydrocortisone (Growth Medium SupplementPack; PromoCell, Germany). The human umbilical vein cell line, EA.hy926 (*ATCC*^®^ CRL2922), was grown in Dulbecco’s Modified Eagle Medium (DMEM; Biochrom, Berlin, Germany) containing 10% FCS (PAA Laboratories, Tiefenbach, Austria), 2 mM L-glutamine, and 50 U/mL each of penicillin and streptomycin.

Cells were incubated with different sFlt-1 concentrations (recombinant human VEGF receptor 1-Fc, R&D Systems, Minneapolis, MN, USA) for different periods of time, as indicated in the Results and Figure legends. ChromPure human IgG-Fc (Jackson ImmunoResearch, Ely, UK) was used as a recombinant control protein. Blockers and inhibitors were pre-incubated for 30 min before stimulation or as indicated in Figure legends: B203580 (10 µM, p38 MAPK-inhibitor; LC Laboratories, Woburn, MA, USA), VEGF (recombinant human VEGF_165_, 50 ng/mL; R&D Systems, Minneapolis, MN, USA).

To determine the role of sFlt-1 on the endothelial stiffness induced by the serum of patients with CKD, we neutralized this protein using a specific antibody and immunoprecipitation as previously described [4]. Sera were obtained from ten patients with CKD (eGFR < 60 mL/min/1.73 m^2^) and four healthy volunteers (eGFR > 60 mL/min/1.73 m^2^) as described elsewhere [5]. As summarized in Table 1, patients were divided into three stages of CKD (Stage 3 CKD: 4 patients; Stage 4 CKD: 3 patients; Stage 5 CKD: 3 patients). Collected samples were stored at −80 °C until they were prepared in batches. Briefly, sera were pre-cleared by adding protein-G Sepharose beads (20 µL per 100 µL serum; GE Healthcare, Danderyd, Sweden) for 2 h at 4 °C. The supernatant was collected after centrifugation (15,000× *g*, 3–4 min) and incubated with monoclonal antibody anti-sFlt-1 (40 µg/mL; R&D Systems, Wiesbaden, Germany) or nonimmune IgG (40 µg/mL) at 4 °C overnight under shaking. After incubation with the primary antibody, protein-G beads (20 µL per 100 µL serum) were added and further incubated for 4 h at 4 °C. The mixture was centrifuged again, and the supernatants were stored at −80 °C until further analysis. EA.hy926 were incubated with control or patients’ sera for 24 h. The protocol was approved by the medical ethical committee of the University Clinics Münster, and written informed consent was obtained from all patients.

### 4.2. Atomic Force Microscopy

Stiffness measurements of the endothelial cell cortex in vitro and ex vivo (isolated mouse aorta) were performed by atomic force microscopy (AFM) nanoindentation technique using a Multimode AFM (Veeco, Munich, Germany) as previously described [52,53].

Experiments were performed on living HUVECs and EA.hy926 cells growing on 15-mm coverslips and treated as described above. For cells treated with the serum of patients, 20–50 values were obtained per patient, given that each value represented a cell/measurement. Mouse aortae were isolated from C57BL/6 mice receiving recombinant mouse sFlt-1 (recombinant mouse VEGF receptor 1-Fc, 300 ng/h diluted in NaCl; R&D Systems, Minneapolis, MN, USA) or control IgG-Fc (IgG2a Fc, 300 ng/h diluted in NaCl; Bio X Cell, Lebanon, NH, USA) for three days by using osmotic minipumps (Alzet model 1007D; Durect Corporation, Cupertino, CA, USA) implanted subcutaneously (s.c.) on the back of each animal [5,39]. Experiments were approved by a governmental committee on animal welfare Landesamt für Natur, Umwelt und Verbraucherschutz Nordrhein-Westfalen (No. 81-02.04.2019.A208) and performed under Germany’s animal protection guidelines. Details about aorta preparation were described elsewhere [53,54,55]. For the experiments, the cells and the aorta preparations were bathed at 37 °C in Hepes-buffered solution (140.0 mM NaCl, 5.0 mM KCl, 1.0 mM MgCl2, 1.0 mM CaCl2, 10.0 mM HEPES, pH 7.4) supplemented with 1% FCS.

### 4.3. Western Blotting Analysis

Confluent cells were *starved overnight* in a serum-free medium and then treated with recombinant sFlt-1 (2 µg/mL) for 10 min. Next, cells were lysed directly in 1× Laemmli buffer containing *β*-Mercaptoethanol, sonicated for 20 min in an ultrasound water bath, and boiled for 5 min at 95 °C. The proteins were subjected to SDS-PAGE (4–20% Mini-PROTEAN TGX Precast Protein Gel; Bio-Rad, Feldkirchen, Germany) and analyzed by Western blotting (Nitrocellulose membrane; GE Healthcare, Munich, Germany) using a rabbit monoclonal antibody against phosphorylated p38 MAPK (Phospho-p38 MAP Kinase (Thr180/Tyr182) (12F8), 1:1000; Cell Signaling, Denver, MA, USA). After incubating the membrane with a mild stripping buffer (low pH glycine solution: 25 mM glycine-HCl, 1% (*w*/*v*) SDS, 0.01% Tween 20, pH 2.2) 6 times for 10 min each and reblocking, the membrane was further incubated with a rabbit polyclonal antibody against (total) p38 MAPK (1:1000; Cell Signaling, USA). To analyze Smad2/Smad3 activation, the membrane was incubated with a rabbit monoclonal antibody against phosphorylated Smad2/Smad3 (1:1000; Cell Signaling, USA) and a rabbit monoclonal antibody against GAPDH (1:1000; Cell Signaling, USA) used as a loading control. After overnight incubation at 4 °C, the membranes were incubated with a secondary antibody coupled to horseradish peroxidase for 1 h at room temperature. Finally, the membrane was exposed to a chemiluminescent substrate (LumiLight Plus; Roche, Mannheim, Germany; Clarity Western ECL Substrate or Clarity Max Western ECL Substrate; Bio-Rad, Hercules, CA, USA) as recommended by the manufacturers for imaging. The signal was recorded with the Azure c600 Ultimate Western Imaging System (Biozym Scientific, Hessisch Oldendorf, Germany). Densitometry analysis was performed using ImageJ software (version 1.53a, National Institute of Health, https://imagej.nih.gov/ij/).

### 4.4. Cellular ROS Detection

Cellular ROS was detected using the fluorescent CellROX™ Deep Red reagent (Assay Kit #C10491, Invitrogen, Waltham, MA, USA) according to the manufacturer’s instructions with minor changes. HUVECs grown in 96-well plates were treated with sFlt-1 or control protein (2 µg/mL) for 30–45 min in the presence or not of inhibitors to induce ROS. Subsequently, 10 µL CellROX™ Deep Red reagent (25 µM in medium) was added to the samples to achieve a final concentration of 2 µM and incubated for 30 min at 37 °C, protected from light. The cells were washed carefully in Hank’s balanced salt solution three times before acquiring fluorescence at 635/665 nm (excitation/emission) using a microplate reader (Tecan Infinite Microplate Reader M200 Pro Tecan; Salzburg, Austria). Cells incubated without CellROX™ Deep Red reagent were used as blank. Tert-Butyl hydroperoxide (TBHP, 200 µM) was used as a positive control.

### 4.5. F-Actin Quantification

F-actin polymerization was quantified as previously described with minor modifications [56]. Cells grown in 96-well plates were fixed with 4% PFA for 15 min at 4 °C and washed with PBS three times for 5 min. After permeabilization with 0.1% Triton X-100/PBS for 5 min at room temperature and washing, Phalloidin-iFluor 594 (1:2000 in PBS; Abcam, Cambridge, UK) and DAPI (10 µg/mL in PBS; Thermo Scientific, Waltham, MA, USA) were added and incubated for 30 min at room temperature. The cells were washed with PBS three times for 5 min, and fluorescence intensity was measured at 590/618 nm for Phalloidin and 360/460 nm (excitation/emission) for DAPI using a microplate reader (Tecan Infinite Microplate Reader M200 Pro Tecan; Salzburg, Austria). Results were calculated by dividing the fluorescent intensity of Phalloidin-594 by the DAPI intensity.

### 4.6. Endothelial Cell Permeability

Transwell permeability assay using streptavidin horseradish peroxidase (HRP) was used to measure bulk (“global”) barrier property without discriminating between transcellular and paracellular transports [56]. HUVECs (25,000 cells/well) were seeded on the top chambers of transwell inserts (0.4 µm pore, 33.6 mm^2^ culture surface, ThinCert^®^ for 24-well plates; Greiner Bio-One, Frickenhausen, Germany) previously coated with gelatin-based coating solution (ready to use; Cell Biologics, Chicago, IL, USA) for 20 min at room temperature. The bottom chambers were filled with growth medium. The next day (24 h after seeding), confluent cell monolayers were treated with recombinant sFlt-1 or control protein for 24 h, as described above. Subsequently, media from both compartments were aspirated, and the top and bottom chambers were refilled with 500 µL medium containing 15 µL Streptavidin-HRP (pre-dilution 1:200; R&D Systems, Minneapolis, MN, USA) or medium only, respectively. After 90-min incubation at 37 °C, three aliquots (20 µL) of media from the lower chamber were transferred to a 96-well plate and incubated with 50 µL of TMB substrate (tetramethylbenzidine, 1:1, stabilized peroxide solution, and stabilized TMB solution; R&D Systems, Minneapolis, MN, USA) for 10 min at room temperature for the reaction to stabilize. The reaction was stopped by adding 25 µL stop solution (2N sulfuric acid; R&D Systems, Minneapolis, MN, USA) into each well. Absorbance was acquired at 450 nm (reference absorbance 540 nm) using a microplate reader (Tecan Infinite Microplate Reader M200 Pro Tecan, Salzburg, Austria).

### 4.7. Real-Time PCR

Total RNA was extracted from HUVECs using the RNeasy Mini Kit (QIAGEN, Hilden, Germany) according to the manufacturer’s instructions. After RNA isolation, reverse transcription of RNA to cDNA (1 µg total RNA in a volume of 20 µL) was performed using the LunaScript RT Master Mix Kit (New England BioLabs, Ipswich, MA, USA) according to the manufacturer’s instructions.

The gene expression was analyzed by real-time PCR using the SYBR Select Master Mix (Applied Biosystems, Darmstadt, Germany) on a CFX Opus 384 Real-Time PCR System (Bio-Rad, Feldkirchen, Germany) with 40 cycles per sample. The cycling temperatures were set at 95 °C for denaturation and 60 °C for annealing and extension. The relative gene expression was analyzed using the 2^−ΔΔCt^ method and 18S as the reference gene. Results were log-transformed before statistical analysis. Human primer sequences are Interleukin-6 forward 5′-acatcctcgacggcatctca-3′ and reverse 5′-caccaggcaagtctcctcatt-3′; Interleukin-8 forward 5′-gtgcagttttgccaaggag-3′ and reverse 5′-ctctgcacccagttttcctt-3′; TGF-β1 forward 5′-gtacctgaacccgtgttgct-3′ and reverse 5′-gtatcgccaggaattgttgc-3′; TGF-β2 forward 5′-ctgctaatgttattgccctcctac-3′ and reverse 5′-cgtgtatccatttccaccctaga-3′; TNF-α forward 5′-ctcttctccttcctgatcgtggca-3′ and reverse 5′-gttggatgtttcgtcctcctcaca-3′; 18S forward 5′-ctcaacacgggaaacctcac-3′ and reverse 5′-cgctccaccaactaagaacg-3′.

### 4.8. Statistical Analysis

Statistical analyses were performed using GraphPad Prism version 9.4.1 for Windows (GraphPad Prism Software Inc., San Diego, CA, USA). In Table 1 (patients’ characteristics), continuous data were presented as median with interquartile range (non-normal distributed) or mean ± standard deviation (SD) (normally distributed data). Due to the relatively small sample size, Kruskal–Wallis test was applied for all comparisons among the three stages of CKD and controls. Controls were excluded from the analysis regarding medication. Regarding dichotomous variables, values were presented as actual numbers (%), and a chi-square test was performed.

For experimental data, Mann–Whitney or Kruskal–Wallis tests, followed by Dunn’s multiple comparisons test, were applied to data with two or more groups, respectively. In Figure 1A, all values (20–50 values/patient) grouped according to the stages of CKD and plotted as box and whiskers were used for comparison among all three stages and controls. Mean values obtained from each patient (dots) were used to compare all CKD patients (Total) and controls. In Figure 1C, all values (boxplot; 20–29 values/mouse) were used for analysis. Mean values obtained from individual mice are represented as dots.

All analyses were considered exploratory. Accordingly, p-values are given as descriptive measures, and the two-sided *p* < 0.05 is considered statistically significant.

## 5. Conclusions

In summary, although the mechanisms underlying endothelial dysfunction are multifactorial and complex, herein, we reinforced the sFlt-1 damaging effects on endothelial cells, which may be of particular interest in the context of CKD and other renal diseases [9]. Determination of endothelial stiffness provides a comprehensive analysis of the status of the endothelial cell structure and function under high sFlt-1 conditions.

## Figures and Tables

**Table 1:** Baseline demographics, clinical characteristics, and risk profile of the study population.

	Controls	CKD Patients				*p*-Value ^1^
		Stage 3 CKD (eGFR 30–59)	Stage 4 CKD (eGFR 15–29)	Stage 5 CKD (eGFR < 15)	Total	
	N = 4	n = 4	n = 3	n = 3	N = 10	
eGFR, mL/min/1.73 m^2^ (mean ± SD)	114 ± 16	52 ± 7	22 ± 5	8 ± 7	30 ± 20	<0.0001
Endothelial dysfunction markers/risk factors						
sFlt-1, pg/mL (median, IQR)	44 (35–108)	101 (65–138)	139 (60–152)	188 (126–574)	133 (72–161)	0.0158
sVCAM-1, ng/mL (median, IQR)	623 (502–710)	845 (794–1007)	1028 (657–1899)	1722 (1008–1924)	1018 (801–1766)	0.0411
vWF, U/mL (mean ± SD)	0.13 ± 0.09	0.56 ± 0.27	0.71 ± 0.33	0.95 ± 0.15	0.76 ± 0.28	0.0575
Aldosterone, ng/dL (median, IQR)	20 (7–44)	8 (4–10)	21 (5–49)	19 (5–49)	10 (5–28)	0.2857
Phosphate, mg/dL (mean ± SD)	3.1 ± 0.48	3.3 ± 0.99	4.0 ± 1.13	4.5 ± 0.51	3.9 ± 0.96	0.0760
Demographic characteristics						
Age, years (mean ± SD)	39 ± 5	72 ± 9	71 ± 4	60 ± 9	68 ± 9	0.0108
Sex, female (%)	33	50	67	33	50	0.7033
Risk factor profile						
Smokers (%)	0	25	67	33	40	0.3589
Hypertension (%)	0	100	100	100	100	0.0046
Diabetes (%)	0	25	67	33	40	0.3589
BMI, kg/m^2^ (mean ± SD)	22 ± 1	26 ± 5	28 ± 6	24 ± 3	26 ± 4	0.1153
Clinical measures						
SBP, mmHg (median, IQR)	120 (120–125)	144 (136–150)	150 (135–150)	140 (130–150)	145 (135–150)	0.0451
DBP, mmHg (median, IQR)	80 (70–80)	81 (76–96)	80 (80–80)	80 (80–85)	80 (80–83)	0.5573
Laboratory values						
Creatinine, mg/dL (mean ± SD)	0.8 ± 0.1	1.1 ± 0.2	2.5 ± 0.6	6.8 ± 2.8	3.3 ± 2.9	<0.0001
HbA1c, % (mean ± SD)	4.6 ± 0.1	5.1 ± 1.0	6.0 ± 1.4	5.3 ± 0.3	5.4 ± 1.0	0.3641
Cholesterol total, mg/dL (median, IQR)	155 (134–178)	193 (180–287)	170 (115–198)	153 (103–177)	177 (144–198)	0.1334
Cholesterol HDL, mg/dL (mean ± SD)	45 ± 3	54 ± 23	48 ± 6	40 ± 6	48 ± 15	0.4853
Cholesterol LDL, mg/dL (mean ± SD)	141 ± 33	129 ± 40	98 ± 67	75 ± 12	103 ± 46	0.1053
Triglycerides, mg/dL (mean ± SD)	152 ± 8	201 ± 49	119 ± 6	194 ± 85	174 ± 62	0.0417
Medication ^2^						
ACE inhibitor, yes (%)	-	100	0	0	40	0.0402
AT1 blocker, yes (%)	-	25	67	33	40	0.1146
Statin, yes (%)	-	25	33	33	30	0.9611
Erythropoietin, yes (%)	-	0	0	0	0	NA
Heparin, yes (%)	-	0	67	67	40	0.1084

^1^ Comparison among the three stages of CKD and controls. ^2^ Controls were excluded from the analysis regarding medication. ACE, angiotensin-converting enzyme; AT1, angiotensin receptor type 1; BMI, body mass index; DBP, diastolic blood pressure; eGFR, estimated glomerular filtration rate; HbA1c, hemoglobin A1c; NA, not applicable; SBP, systolic blood pressure; sFlt-1, soluble Fms-like tyrosine kinase-1; VCAM-1, soluble vascular cell adhesion molecule-1; vWF, von Willebrand factor.

## Data Availability

The original data presented in this study are included in the article. Further inquiries can be directed to the corresponding author.
